# Sex-Dependent Association Between Early Morning Ambulatory Blood Pressure Variations and Acute Mountain Sickness

**DOI:** 10.3389/fphys.2021.649211

**Published:** 2021-03-18

**Authors:** Renzheng Chen, Jie Yang, Chuan Liu, Mengjia Sun, Jingbin Ke, Yuanqi Yang, Yang Shen, Fangzhengyuan Yuan, Chunyan He, Ran Cheng, Hailin Lv, Hu Tan, Xubin Gao, Jihang Zhang, Lan Huang

**Affiliations:** ^1^Institute of Cardiovascular Diseases of PLA, The Second Affiliated Hospital, Third Military Medical University (Army Medical University), Chongqing, China; ^2^Department of Cardiology, The Second Affiliated Hospital, Third Military Medical University (Army Medical University), Chongqing, China

**Keywords:** ambulatory blood pressure, sex difference, morning blood pressure surge, acute mountain sickness, headache, high altitude

## Abstract

**Background:**

Acute high altitude (HA) exposure elicits blood pressure (BP) responses in most subjects, and some of them suffer from acute mountain sickness (AMS). However, a 24-h ambulatory BP (ABP) change and the correlation with the occurrence of AMS in different sexes are still unclear.

**Objectives:**

This prospective study aimed to investigate HA induced BP responses in males and females and the relationship between AMS and 24-h ABP.

**Methods:**

Forty-six subjects were matched according to demographic parameters by propensity score matching with a ratio of 1:1. All the subjects were monitored by a 24-h ABP device; the measurement was one period of 24 h BP. 2018 Lake Louise questionnaire was used to evaluate AMS.

**Results:**

Both the incidence of AMS (14 [60.9%] vs. 5 [21.7%], *P* = 0.007) and headache (18 [78.3%] vs. 8 [34.8%], *P* = 0.003) were higher in females than in males. All subjects showed an elevated BP in the early morning [morning systolic BP (SBP), 114.72 ± 13.57 vs. 120.67 ± 11.10, *P* = 0.013]. The elevation of morning SBP variation was more significant in females than in males (11.95 ± 13.19 vs. −0.05 ± 14.49, *P* = 0.005), and a higher morning BP surge increase (4.69 ± 18.09 vs. −9.66 ± 16.96, *P* = 0.005) was observed after acute HA exposure in the female group. The increase of morning SBP was associated with AMS occurrence (*R* = 0.662, *P* < 0.001) and AMS score (*R* = 0.664, *P* = 0.001). Among the AMS symptoms, we further revealed that the incidence (*R* = 0.786, *P* < 0.001) and the severity of headache (*R* = 0.864, *P* < 0.001) are closely correlated to morning SBP.

**Conclusions:**

Our study demonstrates that females are more likely to suffer from AMS than males. AMS is closely associated with elevated BP in the early morning period, which may be correlated to higher headache incidence in subjects with higher morning SBP.

## Introduction

High altitude (HA) is a hypobaric and hypoxic environment that imposes a formidable physiological challenge for humans. A series of responses in the cardiovascular system and alterations in ventilation allow the human body to acclimatize to hypoxic environments (Hainsworth and Drinkhill, 2007; Parati et al., 2014). Some individuals with poor acclimatization may develop acute mountain sickness (AMS), a syndrome that typically occurs in subjects who ascend to areas that are ≥2500 m above sea level for more than a few hours. AMS is characterized by headache, dizziness, gastrointestinal symptoms, and fatigue, which may rarely progress to fatal high-altitude cerebral edema and/or high-altitude pulmonary edema in severe cases (Luks et al., 2017). Previous studies demonstrated that sex is an independent risk factor for AMS, and lower susceptibility has been reported in males by several studies (Hou et al., 2019).

The elevation of blood pressure (BP) during the day and its decrease at night is a normal physiological phenomenon. Subjects change their state from being asleep to being awake, and start activities in the early morning, which usually leads to an abrupt increase in BP, known as morning BP surge (Sogunuru et al., 2019). Important neurohormonal variations, in particular the activation of the sympathetic nervous system, are involved in this surge (Bilo et al., 2018). Several studies have confirmed that the excessive BP surge during the early morning period is a risk factor for cardiovascular and cerebrovascular events, such as sudden cardiac death and ischemic stroke; these are particularly likely to occur in the early morning (Kario et al., 2003; Metoki et al., 2006; Cheng et al., 2017). Previous studies have reported BP changes when subjects are exposed to hypoxia or HA (Niebauer et al., 2020; Shen et al., 2020). However, no specific information about the relationship between the morning BP surge and AMS has been reported.

Therefore, in our study, we matched demographic parameters to recruit 23 males and 23 females (1:1 ratio). The incidence of AMS and the changes in the 24-h ambulatory BP (ABP) after acute HA exposure were compared in both sexes. Correlation analysis was performed to demonstrate the relationship between changes in BP and AMS. Moreover, we wanted to investigate the potential mechanism of AMS and provide relevant evidence for the prevention and treatment of AMS.

## Materials and Methods

### Study Population and Ethical Considerations

This was a sub-study of a high-altitude prospective cohort study presented in 2019 in Chengdu, China (Shen et al., 2020). All subjects we enrolled were soldiers and 91 were recruited from the ABP study, including 32 females and 59 males. Ten subjects fulfilling any of the following criteria were excluded: severe organic disease, taking any oral medicine, and ABP measurements performed for less than 80% of the whole day. Informed consent was obtained, and all subjects underwent a comprehensive medical examination at low altitude (LA) before the expedition (Chongzhou, 400 m above sea level). After arriving at HA (Litang, 4100 m above sea level), all subjects were asked to perform their usual daily activities. The study was designed on a matched-pair basis. We calculated the propensity scores with the covariates of age, BMI, race, smoking history, alcohol history, and HA living history. A 1:1 nearest-neighbor matching without replacement was performed with a caliper width of 0.10. Forty-six subjects with a 1:1 ratio of males to females were included. The study protocol conformed to the ethical guidelines of the 1975 Declaration of Helsinki, as reflected in a prior approval by the Human Ethics Committee of the Xinqiao Hospital, Third Military Medical University (Identification code, 201907501), and was registered at www.chictr.org.cn (ChiCTR-TRC-No.1900025728).

### Twenty-Four-Hour Ambulatory BP Monitoring

We used an ABP measurement device (Spacelabs 90207, Redmond, WA, United States) to record BP data. Two well-trained cardiovascular physicians were employed and same operators who adopted strict reading criteria and were blind to the subjects’ grouping information performed the examination of ABP monitoring. The BP cuff was applied to the non-dominant arm on a weekday morning and was removed after 24 h to gather the data. All participants were asked to remain still during the measurement. The subjects were instructed to avoid unusual physical activities, and follow a standard schedule at both locations, LA and HA. Day-time and night-time were defined as 6:00–22:00 and 22:00–6:00, respectively (Georgianos and Agarwal, 2015). The recorders measured BP every 30 min during the day-time and every 60 min during the nighttime (Huang et al., 2019).

### Calculation of the Relevant BP Parameters

Pre-waking BP, morning BP, and evening BP were calculated by taking the average BP at 2 h just before the wake-up time (4:00–6:00), at 2 h after the wake-up time (6:00–8:00), and at 2 h before going to bed (22:00–24:00), respectively (Stergiou et al., 2018). The lowest BP was defined as the average of three consecutive readings of the lowest night-time recorded BP. The morning BP surge was defined as the difference between morning BP and the pre-waking systolic BP (SBP). Nocturnal BP fall was calculated as the value of nocturnal decline in BP. The definition of normal BP level and hypertension followed the 2018 Chinese Guidelines for Prevention and Treatment of Hypertension (Joint Committee for Guideline Revision, 2019). BP load was the percentage of BP that exceeded the normal range. The average real variability (ARV) of SBP was calculated by the following formula:

$$\frac{1}{N-1}\,\sum_{K=1}^{N-1}\left|B\,P\,k+1-B\,P\,k\right|$$

where *K* ranges from 1 to N, and N denotes the number of valid BP measurements in the data corresponding to a given subject (Mena et al., 2005). ARVs and ARVd represent the ARV of SBP and diastolic BP (DBP), respectively.

### Assessment of AMS

Subjects ascended from locations at LA to locations at HA within 2 days. The latest Lake Louise questionnaire of AMS was used to assess AMS and was administered in the morning (around 8 h after arriving at HA). Participants completed a questionnaire of four items with the assistance of an experienced doctor. The items included headache, dizziness or lightheadedness, gastrointestinal symptoms, and fatigue. Each item comprised four alternative responses graded from 0 to 3, according to the severity of the symptom (0 for no symptoms, 1 for mild symptoms, 2 for moderate symptoms, and 3 for severe symptoms). AMS was defined as total scores ≥3, with at least 1 point from headache (Roach et al., 2018).

### Statistical Analysis

Continuous variables were presented as mean ± standard deviation. Differences in measurements between males and females with normal distribution were tested using independent-sample *T* test, while the data that did not fit a normal distribution were analyzed by the Mann–Whitney *U* test. Changes in BP and other indexes from LA to HA were compared using a 2 × 2 mixed-model analysis of ANOVA. Categorical data were presented as percentage (%) and were compared by the chi-square test, continuity correction, or Fisher exact test, as appropriate. Spearman correlation coefficients were used to determine the correlation between the different BP index variations after acute HA exposure and AMS, as well as different AMS symptom severity. Statistical significance was assumed at *P* < 0.05. Statistical analyses were performed by SPSS software 26 (IBM, Armonk, NY, United States). Statistical power calculations were performed using the PASS software, version 11 (NCSS, LLC, Kaysville, UT, United States). Results suggested that 46 subjects would provide more than 75% power to detect morning systolic blood pressure (MSBP) and MSBPS differences between subgroups using a two-sided alpha of 0.05.

## Results

### Basic Parameters

There was no significant difference in age, BMI, race, HA living experience, and the history of alcohol and tobacco intake between males and females (Table 1).

**TABLE 1:** Demographic parameters.

Variables	All (*n* = 46)	Female (*n* = 23)	Male (*n* = 23)	*P* value
Age, years	24.96 ± 5.42	24.43 ± 4.51	25.47 ± 6.27	0.520
BMI, kg/m^2^	21.56 ± 1.83	21.43 ± 1.98	21.69 ± 1.72	0.646
Alcohol	0 (0.0%)	0 (0.0%)	0 (0.0%)	1.000
Tobacco	1 (2.2%)	0 (0.0%)	1 (4.3%)	1.000
Han Chinese	46 (100.0%)	23 (100.0%)	23 (100.0%)	1.000
HA living history	0 (0.0%)	0 (0.0%)	0 (0.0%)	1.000

*Values are presented as mean ± standard deviation and percentage (%). BMI, body mass index; HA, high altitude; HA living history was defined as living at a HA for more than 1 year.*

### Effect of Acute High-Altitude Exposure on BP

There were no significant differences in heart rate and SpO_2_ at LA or at HA between the two sexes. The female group had a lower body water percent (55.97 ± 4.31 vs. 60.71 ± 3.66, *P* < 0.001) compared with that of males at the LA location. However, the variation did not reveal a significant difference between the two groups after HA exposure (Table 2).

**TABLE 2:** Effects of acute HA exposure on BP.

Variables	LA			HA			Variation		
	Female (*n* = 23)	Male (*n* = 23)	*P*1 value	Female (*n* = 23)	Male (*n* = 23)	*P*2 value	Female (*n* = 23)	Male (*n* = 23)	*P*3 value
SpO_2_, %	97.21 ± 1.28	96.65 ± 1.82	0.230	86.64 ± 4.00	88.17 ± 2.74	0.135	−10.58 ± 4.01	−8.48 ± 3.65	0.070
Body water percent, %	55.97 ± 4.31	60.71 ± 3.66	<0.001	55.98 ± 4.44	61.18 ± 3.95	<0.001	0.00 ± 1.64	0.48 ± 1.10	0.187
Day-time HR, bpm	75.23 ± 9.11	78.84 ± 6.95	0.138	86.82 ± 6.35	86.58 ± 7.99	0.912	11.59 ± 7.91	7.74 ± 5.55	0.063
Night-time HR, bpm	59.41 ± 10.47	58.04 ± 8.61	0.631	70.97 ± 11.07	66.60 ± 10.49	0.176	11.56 ± 10.97	8.55 ± 8.84	0.312
**BP characteristic, mmHg**									
Day-time SBP	117.15 ± 10.75	125.30 ± 6.50	0.004	123.92 ± 8.00	131.59 ± 10.31	0.007	6.76 ± 10.85	6.29 ± 9.71	0.876
Night-time SBP	105.75 ± 9.92	107.66 ± 9.49	0.568	109.16 ± 11.87	113.83 ± 7.95	0.124	3.41 ± 13.45	6.17 ± 8.31	0.407
Day-time DBP	69.24 ± 4.88	73.73 ± 5.24	0.004	76.45 ± 4.80	79.10 ± 6.34	0.118	7.21 ± 5.23	5.37 ± 7.01	0.318
Night-time DBP	60.58 ± 5.07	59.15 ± 6.69	0.417	65.17 ± 8.55	64.95 ± 7.94	0.926	4.59 ± 8.99	5.80 ± 8.85	0.649
Nocturnal SBP fall, %	9.12 ± 10.63	14.00 ± 7.03	0.074	11.89 ± 7.83	13.12 ± 7.79	0.597	2.77 ± 13.01	−0.88 ± 7.45	0.250
Nocturnal DBP fall, %	12.16 ± 8.84	19.75 ± 6.92	0.002	14.76 ± 9.72	17.69 ± 9.56	0.308	2.60 ± 14.76	−2.06 ± 10.47	0.223
Pre-waking SBP	99.47 ± 9.70	102.54 ± 7.83	0.244	106.74 ± 11.50	112.16 ± 10.56	0.132	7.26 ± 12.33	9.62 ± 10.71	0.637
Pre-waking DBP	56.96 ± 6.30	53.78 ± 5.46	0.075	67.00 ± 10.43	66.66 ± 10.38	0.593	10.04 ± 10.20	13.10 ± 11.09	0.337
Morning SBP	110.87 ± 14.34	118.56 ± 11.83	0.026	122.82 ± 12.05	118.52 ± 9.84	0.192	11.95 ± 13.19	−0.05 ± 14.49	0.005
Morning DBP	67.22 ± 10.3	70.16 ± 8.46	0.296	77.06 ± 10.51	75.41 ± 10.13	0.882	9.84 ± 13.17	5.25 ± 12.68	0.235
Morning SBP surge	11.40 ± 14.38	16.02 ± 15.51	0.240	16.09 ± 13.02	6.36 ± 11.08	0.011	4.69 ± 18.09	−9.66 ± 16.96	0.008
Morning DBP surge	9.14 ± 12.60	16.38 ± 10.28	0.038	10.06 ± 12.09	8.92 ± 10.97	0.257	0.92 ± 13.48	−7.46 ± 12.06	0.031
Evening SBP	117.2 ± 12.99	118.62 ± 12.68	0.708	114.78 ± 15.03	122.18 ± 20.20	0.297	−2.41 ± 18.98	3.56 ± 20.97	0.317
Evening DBP	67.89 ± 9.18	68.94 ± 9.03	0.697	68.89 ± 9.34	67.44 ± 8.90	0.087	1.00 ± 12.67	−1.50 ± 11.17	0.481
Lowest night-time SBP	97.96 ± 12.17	103.25 ± 10.32	0.119	112.40 ± 10.84	116.77 ± 15.84	0.775	14.43 ± 17.59	13.51 ± 18.29	0.863
Lowest night-time DBP	57.09 ± 10.21	55.53 ± 6.61	0.199	60.65 ± 9.21	60.27 ± 7.87	0.279	3.57 ± 14.60	4.75 ± 10.63	0.755
**BP variability, mmHg**									
Day-time ARVs	17.93 ± 5.71	19.61 ± 5.30	0.307	21.05 ± 6.07	20.92 ± 4.82	0.559	3.12 ± 5.50	1.30 ± 5.78	0.282
Night-time ARVs	13.99 ± 7.17	15.02 ± 7.15	0.630	12.80 ± 6.31	11.79 ± 5.20	0.487	−1.20 ± 9.59	−3.23 ± 9.96	0.485
Day-time ARVd	13.89 ± 5.18	15.80 ± 5.11	0.216	16.16 ± 5.60	15.13 ± 5.28	0.393	2.26 ± 7.31	−0.49 ± 8.01	0.199
Night-time ARVd	9.85 ± 9.77	9.81 ± 4.57	0.795	11.08 ± 5.35	9.82 ± 4.47	0.780	1.23 ± 11.52	0.02 ± 6.99	0.669

*Values are presented as mean ± standard deviation. *P*1 and *P*2 were calculated by independent-sample *T* test or Mann–Whitney *U* test; P3 was calculated by 2 × 2 mixed-model analysis of ANOVA. HA, high altitude; LA, low altitude; BP, blood pressure; HR, heart rate; SBP, systolic blood pressure; DBP, diastolic blood pressure; ARVs, average real variability of SBP; ARVd, average real variability of DBP.*

**FIGURE 1:**
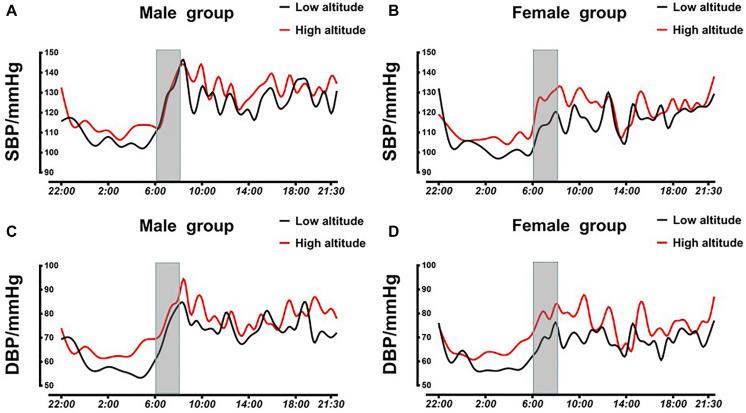
Averaged 24-h SBP and DBP profiles in male and female subjects. **(A)** Averaged 24-h SBP in male subjects at LA and HA. **(B)** Averaged 24-h SBP in female subjects at LA and HA. **(C)** Averaged 24-h DBP in male subjects at LA and HA. **(D)** Averaged 24-h DBP in female subjects at LA and HA. BP, blood pressure; SBP, systolic blood pressure; DBP, diastolic blood pressure; LA, low altitude; HA, high altitude.

Figure 1 shows the 24-h ABP changes at LA and HA both in the female and male groups. Day-time SBP, night-time SBP, day-time DBP, and night-time DBP increased after acute exposure to HA (Figure 1). Furthermore, pre-waking BPs were significantly elevated in both sexes (Figure 1).

Day-time BPs in males were higher than those in females at LA (day-time SBP 117.15 ± 10.75 vs. 125.30 ± 6.50, *P* = 0.004; day-time DBP 69.24 ± 4.88 vs. 73.73 ± 5.24, *P* = 0.004), but variations of day-time BPs changed similarly. There was no obvious difference in nighttime BPs and ARV between the two groups both at LA and at HA (Table 2). The female group showed a lower nocturnal DBP fall, which was associated with a smaller morning DBP surge at LA (nocturnal DBP fall 12.16 ± 8.84 vs. 19.75 ± 6.92, *P* = 0.002; morning DBP surge 9.14 ± 12.60 vs. 16.38 ± 10.28, *P* = 0.038) (Table 2). All subjects underwent a significant increase in MSBP (morning SBP) (114.72 ± 13.57 vs. 120.67 ± 11.10, *P* = 0.013) and morning DBP (68.69 ± 9.44 vs. 76.24 ± 10.24, *P* < 0.001) but not in morning BP surge during the acute HA exposure period (morning SBP surge 13.71 ± 14.97 vs. 11.22 ± 12.93, *P* = 0.374; morning DBP surge 12.76 ± 11.95 vs. 9.49 ± 11.43, *P* = 0.138) (Supplementary Table 1). Interestingly, morning SBP and DBP surge increased in the female group, but reduced in the male group after acute exposure to HA (morning SBP surge 4.69 ± 18.09 vs. −9.66 ± 16.96, *P* = 0.005; morning DBP surge 0.92 ± 13.18 vs. −7.46 ± 12.06, *P* = 0.031) (Table 2). As we further analyzed the BP changes at different time periods between the two groups, we found that this phenomenon could be attributed to a significant elevation of MSBP exhibited in females after acute HA exposure (11.95 ± 13.19 vs. −0.05 ± 14.49, *P* = 0.005) (Table 2).

### AMS and Acute HA Exposure

More cases of AMS were recorded in the females (14 [60.9%] vs. 5 [21.7%], *P* = 0.007), and AMS score was also significantly higher in the female group than in the male group (3.26 ± 2.18 vs. 1.65 ± 1.19, *P* = 0.008). Furthermore, among the AMS symptoms, the incidence of headache was also higher in the female group than in the male group (18 [78.3%] vs. 5 [34.8%], *P* = 0.003) (Table 3).

**TABLE 3:** AMS and related symptoms.

Variables	All (*n* = 46)	Female (*n* = 23)	Male (*n* = 23)	*P* value
AMS	19 (41.3%)	14 (60.9%)	5 (21.7%)	0.007
AMS score	2.46 ± 1.92	3.26 ± 2.18	1.65 ± 1.19	0.008
Headache	26 (56.5%)	18 (78.3%)	8 (34.8%)	0.003
Dizziness	21 (45.7%)	12 (52.2%)	9 (39.1%)	0.375
Gastrointestinal symptoms	21 (45.7%)	13 (56.5%)	8 (34.8%)	0.236
Fatigue	20 (43.5%)	12 (52.2%)	8 (34.8%)	0.234

*Values are presented as mean ± standard deviation or number (%). AMS, acute mountain sickness.*

### Correlation Between BP in the Early Morning and AMS

**FIGURE 2:**
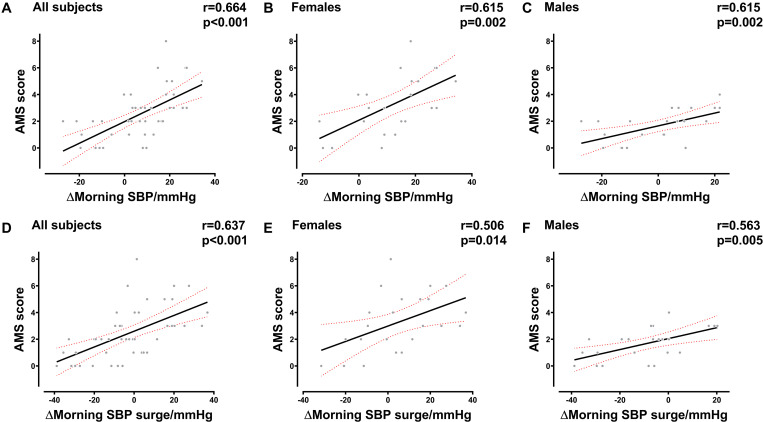
Correlation between the AMS score and BP in the early morning. **(A–C)** Correlation between the AMS score and MSBP variation in all subjects, female subjects, and male subjects. **(D–F)** Correlation between AMS score and morning SBP surge variation in all subjects, female subjects, and male subjects. BP, blood pressure; SBP, systolic blood pressure; MSBP, morning systolic blood pressure; AMS, acute mountain sickness. Δ, variation after acute HA exposure.

**FIGURE 3:**
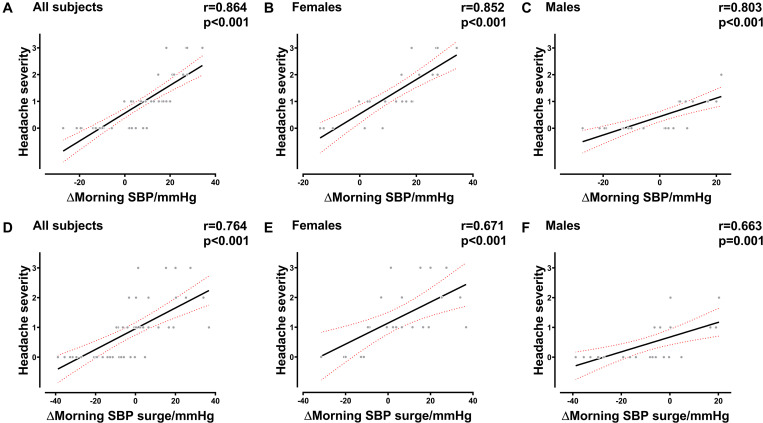
Correlation between the severity of headache and BP in the early morning. **(A–C)** Correlation between the severity of headache and MSBP variation in all subjects, female subjects, and male subjects. **(D–F)** Correlation between the severity of headache and morning SBP surge variation in all subjects, female subjects, and male subjects. BP, blood pressure; SBP, systolic blood pressure; MSBP, morning systolic blood pressure. Δ, variation after acute HA exposure.

The results of the correlation analysis indicated that there was a strong correlation between AMS and morning SBP surge variations in females (*R* = 0.584, *P* = 0.003), males (*R* = 0.588, *P* = 0.003), and all subjects (*R* = 0.659, *P* < 0.001). The variation of MSBP was also significantly correlated with AMS in females (*R* = 0.558, *P* = 0.006), males (*R* = 0.652, *P* = 0.001), and all subjects (*R* = 0.662, *P* < 0.001) (Supplementary Table 2). Besides, we found that the variation in MSBP was strongly correlated with AMS score in the total study population (Figure 2A, *R* = 0.664, *P* < 0.001), the female group (Figure 2B, *R* = 0.615, *P* = 0.002), and the male group (Figure 2C, *R* = 0.615, *P* = 0.002). The variation of morning SBP surge was significantly correlated with AMS score in the female group (Figure 2E, *R* = 0.506, *P* = 0.014), male group (Figure 2F, *R* = 0.563, *P* = 0.005) and all subjects (Figure 2D, *R* = 0.637, *P* < 0.001). Furthermore, we demonstrated that MSBP and morning SBP surge have stronger correlations with the severity of headache in females (Figure 3B, *R* = 0.852, *P* < 0.001) (Figure 3E, *R* = 0.671, *P* < 0.001), males (Figure 3C, *R* = 0.803, *P* < 0.001) (Figure 3F, *R* = 0.663, *P* = 0.001), and all subjects (Figure 3A, *R* = 0.864, *P* < 0.001) (Figure 3D, *R* = 0.764, *P* < 0.001).

## Discussion

To our knowledge, this study was the first to investigate the changes of ABP in males and females after acute HA exposure. In this study, we showed the changes of ABP after acute HA exposure in both sexes and analyzed the correlation between ABP and AMS. Interestingly, we found that females exhibited a remarkable elevation in BP during the early morning period as well as an increase of the morning BP surge. However, morning BP surge declined in males, which may be attributed to an inapparent increase of MSBP after acute HA exposure. The BP readings of female participants were on average lower than those of the male participants. Furthermore, we found that there was a significant correlation between SBP in the early morning and AMS. Besides, in order to explain this phenomenon, we further revealed that MSBP was significantly correlated with the severity of headache among the AMS symptoms in males, females, and all subjects.

### AMS and Sex Differences

Previous studies have demonstrated that 10–70% of travelers experience various degrees of AMS when ascending to elevation of ≥2500 m. The rate of AMS in our study was 41.5%, which is consistent with those reported previously (Ren et al., 2010). AMS was diagnosed with non-specific symptoms that occur at altitudes of ≥2500 m in unacclimatized subjects, within usually a few hours after arrival at a location with HA. The progression of AMS can rarely lead to fatal HA cerebral edema in severe cases (Bärtsch and Swenson, 2013). Previous reports verified the factors, including age, the speed of ascent, arrival altitude, and the ethnicity of the enrolled study subjects, that are associated with AMS (Luks et al., 2017).

It is always a controversial topic to discuss the relationship between sex difference and AMS as shown in previous studies. A cohort study showed that females developed AMS at a rate of 69.2%, which was consistent with our results (Boos et al., 2018). However, there were also studies that failed to show a difference in the rate of AMS between the two sexes (Pesce et al., 2005; Gonggalanzi et al., 2016). These inconsistent findings might be attributed to the ethnic and age differences, history of HA exposure, and prophylactic usage of drugs for high-altitude sickness. Several factors could be considered for the higher incidence of AMS in females. First, reporting bias can be a major contributing factor. Soldiers often undertake cardiopulmonary fitness training. Results from a sample from a military population might have different findings than a civilian sample. Furthermore, previous studies have also found that females have a large oxygen saturation reduction both after HA exposure and exercise at LA, and larger oxygen saturation reduction after exercise at LA was an independent risk for AMS upon ascent (Dominelli et al., 2017; Shen et al., 2020). Women are also more susceptible to hypoxemia, which may explain the higher incidence of AMS. In our study, the reduction of oxygen saturation was less in males, but the difference did not reach a statistical significance. Besides, 17 beta-estradiol could reduce the operating point for osmoregulation of arginine vasopressin, and contribute to fluid retention, which could increase BP (Stachenfeld et al., 1999). Interestingly, there was no observed increase in elevation of total body water in females after acute HA exposure compared with that in males. This suggests that some other hormone may be associated with fluid balance retention after ascent to a location at HA. Furthermore, in a study of a small female population, results indicated that anxiety might partly explain the difference in AMS susceptibility when compared to males (Boos et al., 2018). While there was still contradictory conclusion, this may be speculation and we did not perform the relevant psychological investigations (Niedermeier et al., 2017).

### Significance and Mechanism of Excessive Morning BP Elevation

Previous studies have demonstrated a correlation between exaggerated morning BP surge and adverse cardiovascular events in patients with hypertension (Stachenfeld et al., 1999). Higher morning SBP has been demonstrated to be associated with arterial stiffness (Suh et al., 2014), which indicates that higher elevated BP in the early morning period is also harmful for a healthy population. Several physiological mechanisms might be involved in an excessive morning BP surge. The most important factor was the excessive activation of the sympathetic nervous system, which is accompanied by the activation of the release of norepinephrine and adrenaline, in the early morning period (Dodt et al., 1997). The levels of renin, angiotensin II, and plasma aldosterone were also higher during the day-time than during the nighttime. These changes as well as fluid retention lead to an elevation of arterial BP. Furthermore, endothelial function damage (Otto et al., 2004), reduced sympathetic baroreflex (Otto et al., 2004) and the level of salt loading (Osanai et al., 2000) could also be involved, considering the current belief that systematic cardiovascular responses are related to enhanced sympathetic nerve activity and the release of catecholamines caused by acute altitude hypoxia (Mazzeo and Reeves, 2003). Thus, BP variations in the early morning at HA are probably affected by sympathetic activation. We also hypothesize that excessive early morning BP could have adverse effects after acute HA exposure.

### Association Between Morning BP Surge and AMS

Previous studies did not demonstrate a significant difference in early morning BP surge between men and women at LA (Sogunuru et al., 2019). In our study, the morning DBP surge was slightly higher in males than in females at LA. This might be attributable to damage of endothelium-dependent vasodilation due to a higher rate of smoking in males than in females (Motoyama et al., 1997). Furthermore, few studies have reported the correlation between the early morning BP change in plateau environment among males and females. In our investigation, we revealed that morning BP surge was higher in the female group, while it declined in males after acute HA exposure. Female subjects showed a higher increase of MSBP than males did; however, differences in sex hormones could affect MSBP by influencing body fluid regulation. The changes that we observed in the body fluids did not match that hypothesized, indicating that some other factors may be involved. As the excessive activation of the sympathetic nervous system in the early morning seemed to be the primary reason for BP surge and females are more susceptible to hypoxemia, the difference in cardiovascular responses induced by sympathetic activation and sensitivity toward hypoxia may be a probable cause (Iturriaga, 2018). But some indexes such as nocturnal desaturation and time course, which could be better for representing the data of oxygen saturation, were not measured. As to the association between morning BP surge and AMS, a recent study found that a transient autonomic dysfunction resulted in a more pronounced BP drop during initial hypoxic exposure in AMS subjects, which suggested a relationship between autonomic stimulation and AMS (Niebauer et al., 2020). While hypoxia-induced sympathetic overactivation might also be viewed as a possible cause of autonomic dysfunction. Our linear analysis showed that the increase of morning BP surge as well as BP in early morning period were strongly correlated with AMS. Moreover, there was also a stronger correlation in MSBP and the severity of headache. The development of headaches in AMS may be induced by the elevation of BP in the early morning period after HA exposure, because an excessive rise in BP might result in symptoms, such as dizziness and headaches. Thus, we supposed that there might be a higher sensitivity toward hypoxia, leading to a higher morning BP elevation induced by sympathetic activation in HA environments, which is associated with AMS in females.

### Early Morning Surge of BP and AMS Prevention

Our findings could be helpful and shed light on the prevention of AMS. Females could be more in need of preventive treatment compared with males. Moreover, treatments that specifically target the morning BP surge may be worthy of further investigation. Besides, previous studies point that the usage of diuretics, such as acetazolamide (Lipman et al., 2019), to improve fluid retention levels could decrease the incidence of AMS. Although a previous small cohort study showed that reduction in sympathetic excitability by use of beta blockers at altitude was not favorable for BP reduction (Faulhaber et al., 2003), the efficacy of drug therapy in decreasing morning BP surge for as a treatment for AMS still needs to be verified by larger cohort studies.

### Limitations

There are several limitations in our present study. First, the number of subjects was relatively small; a larger cohort study needs to be performed to verify the results. Second, we did not examine parameters related to the biochemical and cerebral blood flow as well as some indexes, which could more thoroughly explore oxygen saturation, due to limitations of HA conditions in this study. The potential mechanism underlying this phenomenon remains to be studied. Moreover, age-related reference ranges for morning BP surges are not currently available. Furthermore, the classification of AMS was based on a self-report without immediate medical control, which did lead to possible classification bias especially in different genders. Besides, the study subjects were mostly young Chinese people from the Han population. With a narrow study population, the application of our findings might be limited.

## Conclusion

Our study demonstrated that morning BP surge increased more in females compared with males after acute exposure to HA, which may be related to an elevation of MSBP. AMS score strongly correlated with morning BP surge and MSBP in the correlational analyses. Furthermore, headache, a typical symptom of AMS, showed a higher correlation with morning BP surge and MSBP compared with other AMS symptoms. To our knowledge, this is the first study to investigate the relationship of ABP variations and AMS following acute exposure to HA, especially in males and females. However, the exact mechanisms involved need to be further elucidated, and larger cohort studies are also needed to verify the results.

## Data Availability Statement

The raw data supporting the conclusions of this article will be made available by the authors, without undue reservation.

## Ethics Statement

The studies involving human participants were reviewed and approved by the Human Ethics Committee of the Xinqiao Hospital, Third Military Medical University (Identification code, 201907501). The patients/participants provided their written informed consent to participate in this study.

## Author Contributions

ReC, JY, and LH worked on the conception of the study. ReC, MS, YY, YS, JK, FY, CH, RaC, and HL contributed to the data collection. CL and JY checked the data. RaC, YS, and MS performed the statistical analysis. JY and ReC drafted the manuscript. XG, CL, HT, JZ, and LH reviewed the manuscript. All authors read and approved the final version of the manuscript.

## Conflict of Interest

The authors declare that the research was conducted in the absence of any commercial or financial relationships that could be construed as a potential conflict of interest.
