# Supplementary material for: Sex-Dependent Association Between Early Morning Ambulatory Blood Pressure Variations and Acute Mountain Sickness
Source: Front Physiol. 2021 Mar 18;12:649211. doi: 10.3389/fphys.2021.649211 (PMC8012890; doi:10.3389/fphys.2021.649211)
Supplement: Supplementary file 1 [file Data_Sheet_1.docx]

Supplementary Material

**Supplement table 1. Effect of acute HA exposure on BP in the total population.**

| **Variables** | **LA** | **HA** | **Variation** | **HA vs. LA** |
| --- | --- | --- | --- | --- |
|  | **Total (n23)** | **Total (n=23)** | **Total (n=46)** | **P value** |
| SpO_2_, % | 96.93±1.58 | 87.40±3.48 | -9.53±3.94 | <0.001 |
| Body water percent, % | 58.34±4.62 | 58.59±4.92 | 0.24±1.40 | 0.250 |
| day-time HR, bpm | 77.03±8.22 | 86.70±7.14 | 9.66±7.03 | <0.001 |
| night-time HR, bpm | 58.73±9.50 | 68.78±10.89 | 10.06±9.97 | <0.001 |
| **BP characteristic, mmHg** |  |  |  |  |
| day-time SBP | 121.23±9.7 | 127.76±9.92 | 6.53±10.19 | <0.001 |
| night-time SBP | 106.71±9.65 | 111.5±10.26 | 4.79±11.14 | 0.006 |
| day-time DBP | 71.48±5.50 | 77.78±5.72 | 6.29±6.18 | <0.001 |
| night-time DBP | 59.87±5.91 | 65.06±8.16 | 5.19±8.84 | <0.001 |
| nocturnal SBP fall, % | 11.56±9.25 | 12.51±7.75 | 0.94±10.65 | 0.550 |
| nocturnal DBP fall, % | 15.96±8.74 | 16.23±9.65 | 0.27±12.87 | 0.888 |
| Pre-waking SBP | 101.01±8.85 | 109.45±11.25 | 8.44±11.48 | <0.001 |
| Pre-waking DBP | 55.37±6.05 | 66.94±10.29 | 11.57±10.65 | <0.001 |
| Morning-SBP | 114.72±13.57 | 120.67±11.10 | 5.95±14.98 | 0.013 |
| Morning-DBP | 68.69±9.44 | 76.24±10.24 | 7.55±12.99 | <0.001 |
| Morning SBP surge | 13.71±14.97 | 11.22±12.93 | -2.49±18.79 | 0.350 |
| Morning DBP surge | 12.76±11.95 | 9.49±11.43 | -3.27±13.34 | 0.138 |
| Evening-SBP | 117.91±12.71 | 118.48±18 | 0.57±20.01 | 0.952 |
| Evening-DBP | 68.42±9.02 | 68.17±9.05 | -0.25±11.88 | 0.887 |
| Lowest night-time SBP | 100.61±11.48 | 114.58±13.6 | 13.97±17.75 | <0.001 |
| Lowest night-time DBP | 56.31±8.54 | 60.46±8.48 | 4.16±12.64 | <0.001 |
| **BP variability, mmHg** |  |  |  |  |
| Day-time ARVs | 18.77±5.51 | 20.98±5.42 | 2.21±5.66 | 0.011 |
| Night-time ARVs | 14.51±7.10 | 12.30±5.74 | -2.21±9.73 | 0.130 |
| Day-time ARVd | 14.85±5.18 | 15.64±5.41 | 0.80±7.68 | 0.438 |
| Night-time ARVd | 9.83±7.54 | 10.45±4.91 | 0.62±9.44 | 0.221 |

Values are presented as mean ± standard deviation.

HA: high altitude; BP: blood pressure; HR: heart rate; SBP, systolic blood pressure; DBP: diastolic blood pressure; ARVs: average real variability of SBP; ARVd: average real variability of DBP; LA: low altitude; HA: high altitude.

**Supplement table 2. Correlations between AMS symptoms and BP in the early morning.**

| **Variables** | **All subjects (n=46)** | | **Female (n=23)** | | **Male (n=23)** | |
| --- | --- | --- | --- | --- | --- | --- |
|  | **MSBP** | **MSBPS** | **MSBP** | **MSBPS** | **MSBP** | **MSBPS** |
| AMS | 0.662*** | 0.659*** | 0.558** | 0.584** | 0.652** | 0.588** |
| AMS score | 0.664*** | 0.637*** | 0.615** | 0.506* | 0.615** | 0.563** |
| Headache | 0.786*** | 0.750*** | 0.652** | 0.715*** | 0.785*** | 0.647** |
| Headache severity | 0.864*** | 0.764*** | 0.852*** | 0.671*** | 0.803*** | 0.663** |
| Dizziness | 0.263 | 0.181 | 0.295 | 0.177 | 0.202 | 0.054 |
| Dizziness severity | 0.285 | 0.203 | 0.316 | 0.182 | 0.202 | 0.054 |
| Gastrointestinal symptoms | 0.095 | 0.207 | 0.060 | 0.212 | 0.000 | 0.076 |
| Gastrointestinal symptoms severity | 0.160 | 0.242 | 0.151 | 0.211 | 0.000 | 0.076 |
| Fatigue | 0.135 | 0.268 | 0.039 | 0.236 | 0.110 | 0.220 |
| Fatigue severity | 0.164 | 0.267 | 0.097 | 0.211 | 0.129 | 0.247 |

Values are Spearman correlation coefficient. *: P<0.05; **: P<0.01; ***: P<0.001.

BP: blood pressure; AMS: acute mountain sickness; MSBP: morning systolic blood pressure; MSBPS: morning systolic blood pressure surge.
